# Clinical feasibility test of 60 kVp double-low-dose coronary CT angiography with a deep learning reconstruction algorithm

**DOI:** 10.1186/s13244-026-02223-6

**Published:** 2026-02-10

**Authors:** Xi Wu, Manman Zhu, Yixuan Zou, Jialin Luo, Weiling He, Wenjie Sun, Hui Shi, Peng Liu, Feng Huang

**Affiliations:** 1 Department of Radiology, Hunan Provincial People’s Hospital (The First Affiliated Hospital of Hunan Normal University), Changsha, China; 2 United Imaging Healthcare, Shanghai, China

**Keywords:** Coronary CT angiography, low-dose, deep learning reconstruction

## Abstract

**Objectives:**

To test the feasibility of 60 kVp double-low-dose coronary CT angiography (CCTA) with a deep learning reconstruction (DLR) algorithm.

**Materials and methods:**

Eighty-nine patients (44 females, 59.9 ± 13.2 years, 23.1 ± 3.3 kg/m^2^) with known or suspected coronary artery disease were prospectively enrolled. Each patient underwent the double-low-dose CCTA (60-kVp, 28 mL contrast at 2.5 mL/s) and was immediately followed by routine-dose CCTA (100-kVp, 44 mL contrast at 4.0 mL/s). Routine-dose data were reconstructed using hybrid iterative reconstruction (RD-HIR), and double-low-dose data were reconstructed using both HIR (LD-HIR) and DLR (LD-DLR). The consistency of both coronary stenosis assessments and CT-derived fractional flow reserve (CT-FFR) values between low-dose and routine-dose images was quantified using receiver operating characteristic analysis at various levels. Segment-level image quality scores (IQS), signal-noise-ratio (SNR), and contrast-noise-ratio (CNR) were compared among three groups.

**Results:**

Double-low-dose CCTA achieved a significant reduction in both radiation dose (0.60 ± 0.12 mSv vs 4.43 ± 1.42 mSv) and contrast volume compared to routine-dose CCTA. For the per-segment level, LD-DLR showed significantly higher specificity (0.99 vs 0.94), positive predictive value (0.91 vs 0.68), and accuracy (0.98 vs 0.94) for ≥ 50% coronary stenosis compared to LD-HIR. The area under the curve of LD-DLR was significantly higher than LD-HIR for ≥ 50% stenosis at per-segment (0.94 vs 0.92), per-vessel (0.92 vs 0.89), and per-patient (0.92 vs 0.85) levels; and for CT-FFR ≤ 0.80 at per-vessel (0.94 vs 0.74), LAD-vessel (0.94 vs 0.71), and LCX-vessel (0.99 vs 0.67) levels. The IQS, SNR, and CNR of LD-DLR were not inferior to those of RD-HIR in all segments.

**Conclusions:**

The 60 kVp double-low-dose CCTA with DLR can significantly reduce radiation dose and simultaneously maintain the high consistency of coronary stenosis and CT-FFR assessments with routine-dose CCTA.

**Critical relevance statement:**

The 60 kVp double-low-dose CCTA protocol is feasible with a novel DLR algorithm without compromising the performance of coronary stenosis and CT-FFR assessments.

**Key Points:**

- Is a 60 kVp double-low-dose CCTA protocol with a DLR algorithm feasible for routine clinical application?
- The 60 kVp CCTA protocol with the DLR algorithm reduced radiation dose by 86.5% and contrast dose by 36.4%.
- The 60 kVp CCTA with DLR achieved high consistency of coronary stenosis and CT-FFR values with the routine-dose 100 kVp CCTA.

**Graphical Abstract:**

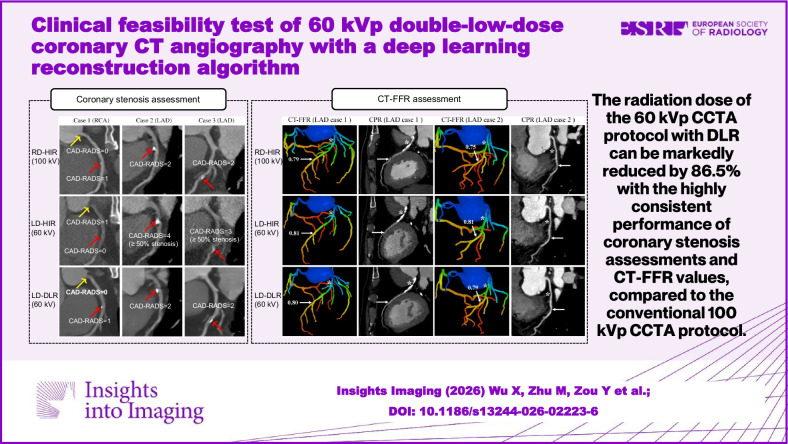

## Introduction

Coronary computed tomography angiography (CCTA) has become a widely adopted non-invasive imaging modality for rapid diagnosis of coronary artery disease (CAD), providing detailed visualization of coronary anatomy and stenosis assessment [1, 2]. Its high diagnostic accuracy is well-established, as demonstrated by Kumar et al, CCTA achieved high sensitivity and negative predictive value (NPV) (both > 95%) while maintaining excellent specificity for CAD detection [3]. Furthermore, a study from Machado et al showed that 77% of patients with stable chest pain could avoid invasive coronary angiography (ICA) following CCTA evaluation [4].

Given the trend toward earlier onset of CAD, CCTA is being performed on a large and growing patient population [5], which raises concerns regarding the potential long-term risk of radiation-induced carcinogenesis [6]. A particular concern for patients requiring repeated follow-up CCTA scans, such as those with established CAD or a history of intervention. Additionally, the administration of iodinated contrast medium carries a risk of contrast-induced nephropathy, especially in patients with pre-existing renal impairment [7, 8]. Consequently, achieving a “double-low” strategy—substantially reducing both radiation and contrast doses while maintaining uncompromising image quality—has become a primary objective in current CCTA research [9–11].

To overcome the inherent increase in image noise associated with the double-low strategy, the advanced deep learning reconstruction (DLR) algorithm has been widely utilized to improve CCTA image quality. For example, Caruso et al applied an 80 kVp CCTA protocol with DLR in non-obese patients, yielding a mean effective dose (ED) of 2.36 mSv with a 42% reduction [9]. Zhu et al explored the feasibility of a 70 kVp protocol with DLR in normal-weight and overweight groups, achieving reduced mean EDs of 1.36 mSv and 1.59 mSv [10]. And Li et al further reported that a 70 kVp protocol combined with DLR achieved a mean ED of 0.75 mSv with a 54.5% reduction for patients with body mass index (BMI) ≤ 26 kg/m^2^ [11].

Nevertheless, the lowest tube voltage currently available in clinical practice is 60 kVp, and the effectiveness of a 60 kVp protocol combined with a low-dose contrast agent for coronary imaging remains unreported. Therefore, this study aimed to test the feasibility of a 60 kVp double-low-dose CCTA protocol with a novel DLR algorithm and further explore its performance of coronary stenosis and CT-derived fractional flow reserve (CT-FFR) assessments, using the conventional 100 kVp CCTA protocol with the hybrid iterative reconstruction (HIR) algorithm as the reference.

## Materials and methods

### Participant enrollment

This study was approved by the Medical Ethics Committee of Hunan Provincial People’s Hospital (No. [2024]-46). From July 2024 to August 2025, 115 consecutive patients scheduled for CCTA due to known or suspected CAD were prospectively enrolled, and written informed consent was obtained from all participants. Exclusion criteria included nephropathy (*n* = 3), presence of coronary stents (*n* = 6), history of bypass grafting (*n* = 2), incomplete raw data (*n* = 8), and failure to obtain CT-FFR results (*n* = 7). All reconstructed CCTA images were reviewed to exclude those deemed non-diagnostic due to high heart rate (HR) or arrhythmia. Ultimately, 89 patients were included for further evaluation and analysis.

The required sample size was calculated using G*Power software (version 3.1.9.7). Based on a study design involving the same group of patients undergoing three repeated measurements, a priori sample size estimation was performed with a statistical power of 0.95 and a significance level of 0.05. Assuming a medium effect size (*f* = 0.25), the calculation indicated that a minimum of 43 participants were needed.

### CCTA acquisition

All CCTA examinations were performed using a 320-row detector scanner (uCT 960+, United Imaging Healthcare) in a prospective ECG-triggered axial mode, with data acquisition phase set at 30–80% of the R-R interval. Prior to scanning, all participants received breath-hold training and were administered 0.5 mg of sublingual nitroglycerin. Each participant first underwent a low-dose scanning, immediately followed by a routine-dose scanning. The low-dose protocol employed a tube voltage of 60 kVp, with a contrast agent (Iomeprol, 400 mg I/mL) injection rate of 2.5 mL/s and a total volume of 28 mL. The routine-dose protocol used a tube voltage of 100 kVp, with a contrast injection rate of 4.0 mL/s and the total volume of 44 mL.

The remaining acquisition parameters were identical between these two protocols: 280 × 0.5 mm collimation, 0.25 s rotation time, and a reference exposure of 230 mAs. The prospective ECG-gated scanning was performed using a threshold-triggering technique with a region of interest (ROI) in the descending aorta. The scan was automatically initiated 2 s after the ROI attenuation signal reached a preset trigger threshold of 180 HU. For both protocols, the volume CT dose index (CTDIvol) and dose-length product (DLP) were recorded. The ED was calculated by multiplying the DLP by a chest-specific conversion coefficient *k* = 0.014 mSv × mGy^−1^ × cm^−1^.

### Image reconstruction and post-processing

After data acquisition, the optimal reconstruction phase within the full cardiac cycle was automatically selected using the artificial intelligence-based ePhase algorithm (United Imaging Healthcare) to improve image quality. The routine-dose CCTA data were reconstructed with a medium-high level HIR algorithm (Karl 3D, United Imaging Healthcare), shortened as Routine-dose CCTA data reconstructed by the HIR algorithm (RD-HIR) group. The low-dose CCTA data were reconstructed using both the same medium-high level HIR and a vendor-specific DLR algorithm (CardioBoost, United Imaging Healthcare) with a medium denoising level, termed as Low-dose CCTA data reconstructed with the HIR algorithm (LD-HIR) and Low-dose CCTA data reconstructed with the DLR algorithm (LD-DLR) groups. HIR employed a soft kernel, while DLR, which was not configured with a similar kernel setup, used an algorithm-embedded image representation with a soft option. To minimize motion artifacts, the artificial intelligence-based motion correction technique (CardioCapture, United Imaging Healthcare) with medium-high strength was applied in all three groups. All images were reconstructed with a 512 × 512 matrix, a slice thickness of 0.5 mm, and a slice interval of 0.5 mm, which were subsequently assessed on a dedicated clinical workstation (uWS-CT, United Imaging Healthcare).

### Coronary stenosis assessment

Two radiologists (with seven years of experience in cardiac radiology), who were blinded to the reconstruction algorithms and patient information, independently evaluated the severity of stenosis in each coronary segment. Image series of RD-HIR, LD-HIR and LD-DLR groups were mixed and presented to radiologists in a random order to minimize assessment bias. The right coronary artery (RCA) and left anterior descending (LAD) were divided into proximal, mid, and distal segments, while the left circumflex (LCX) was divided into proximal and distal segments [12]. Multi-planar reconstruction was utilized to analyze each segment in at least two orthogonal planes, with additional planes applied when necessary. Both radiologists were permitted to freely adjust the display window setting and zoom the images to optimize the assessment of stenosis.

At the segment level, stenosis was classified as positive (moderate or severe) if the lumen diameter reduction was ≥ 50%, and as negative (mild) if it was < 50%. This definition was then aggregated upward: vessel-level stenosis was considered positive if any segment within the coronary artery was positive, and patient-level stenosis was positive if any major coronary artery was positive. Furthermore, stenosis was evaluated in detail using the Coronary Artery Disease Reporting and Data System (CAD-RADS) [13], with each segment assigned a corresponding score (0–5 points). The vessel-level CAD-RADS score was determined by the maximum score among its segments, and the patient-level score by the maximum across the three major vessels.

In instances where the two primary radiologists disagreed in their assessments, a third senior radiologist with 12 years of experience in cardiac radiology was consulted to adjudicate and reach a final consensus. The consistency of coronary stenosis between low-dose and routine-dose groups was subsequently analyzed. Given that low-kVp acquisitions can exacerbate blooming artifacts from dense calcifications and potentially lead to overestimation of stenosis, this study also included a preliminary subgroup analysis focusing on patients with a high coronary artery calcium score (CACS), defined as an Agatston score > 400 [14].

### CT-FFR assessment

CT-FFR values of RCA, LAD, and LCX vessels in three groups were measured using the CT-FFR Coronary software in the uOmnispace processing system (United Imaging Healthcare). The derivation of CT-FFR values from CCTA data followed an established four-step computational process [15]: anatomic model reconstruction, centerline definition, boundary condition, and finally, CT-FFR calculation. CT-FFR values were measured at a location 20 mm distal to the stenosis in vessel segments with a diameter of at least 2 mm. Lesions were classified as ischemic (positive) if CT-FFR ≤ 0.80, and non-ischemic (negative) if CT-FFR > 0.80. The consistency of CT-FFR values between low-dose and routine-dose CCTA images was subsequently evaluated.

### Image quality assessment

The subjective image quality score (IQS) for each segment was further independently and blindly assessed by the two radiologists mentioned above using a 5-point Likert scale. The scoring criteria were defined as follows: 5 (excellent lumen enhancement with clear vessel boundary delineation), 4 (good lumen enhancement with mostly clear boundary delineation), 3 (acceptable lumen enhancement with suboptimal vessel boundary delineation), 2 (inadequate lumen enhancement with obvious vessel blurring), and 1 (poor lumen enhancement with severe vessel blurring).

The objective image quality, including signal-to-noise-ratio (SNR) and contrast-to-noise-ratio (CNR), was evaluated for each segment. The third radiologist independently performed three repeated measurements on manually selected ROIs. The formulas used were: SNR =${\mu }_{{{\mathrm{seg}}}}/{\sigma }_{{{\mathrm{seg}}}}$, CNR = $({\mu }_{{{\mathrm{seg}}}}-{\mu }_{{{\mathrm{fat}}}})/{\sigma }_{{{\mathrm{fat}}}}$, where ${\mu }_{{{\mathrm{seg}}}}$ and ${\mu }_{{{\mathrm{fat}}}}$ are mean CT values of the vessel segment and fat tissue, and ${\sigma }_{{\mathrm{seg}}}$ and ${\sigma }_{{\mathrm{fat}}}$ represent their standard deviations (SDs). Segment ROIs (2–5 mm in diameter) were placed in the largest cross-sectional area while avoiding calcified plaques. Fat ROIs (∼5 mm in diameter) were positioned within chest fat at the aortic root level.

The mean IQS from two readers and the average SNR and CNR of three repeated measurements were subsequently compared among three groups.

### Statistical analysis

All statistical analyses were performed using Python software (version 3.9) and R software (version 4.4.2). The Shapiro-Wilk test was used to check the normality of the data. The CTDIvol, DLP, and ED between the routine-dose and low-dose protocols were compared with the Wilcoxon signed rank test.

The consistency of coronary stenosis assessments and CT-FFR values between two low-dose CCTA and routine-dose CCTA groups was checked with receiver operating characteristic (ROC) analysis. The area under the curve (AUC) values of LD-HIR and LD-DLR were compared using DeLong’s test. The sensitivity, specificity, and accuracy between LD-HIR and LD-DLR were compared using McNemar’s test, while the positive predictive value (PPV) and NPV were compared using either the Chi-Square test or Fisher’s Exact test, as appropriate. Furthermore, the 95% confidence intervals (CIs) of sensitivity, specificity, accuracy, PPV, and NPV were adjusted based on the Generalized Estimating Equations (GEE) models to account for potential within-subject correlations [16].

The Friedman test was used to analyze differences in IQS, SNR, and CNR among RD-HIR, LD-HIR, and LD-DLR groups, and the post-hoc test adopted Dunn’s test with Bonferroni correction for comparison between any two groups. In addition, detailed stenosis assessment based on CAD-RADS scores was analyzed using the weighted Cohen’s kappa test, with 95% CIs and significance testing performed via the Bootstrap method [17]. Inter-rater agreement for IQS was also assessed using the weighted Cohen’s kappa test, where kappa values ≤ 0.20, 0.21–0.40, 0.41–0.60, 0.61–0.80, and > 0.81 indicate poor, fair, moderate, good, and excellent agreement, respectively. A *p* value < 0.05 or an adjusted *p* value < 0.05 was considered statistically significant.

## Results

### Demographic characteristics and radiation dose

A total of 89 participants (44 females; mean age: 59.9 ± 13.2 years; mean BMI: 23.1 ± 3.3 kg/m^2^) were included in the final analysis. Detailed demographic characteristics are presented in Table S1. Compared with the routine-dose protocol, the low-dose CCTA protocol resulted in a substantial 86.5% reduction in radiation exposure, with significantly lower dose metrics: CTDIvol (22.6 ± 7.2 vs 3.0 ± 0.6 mGy, *p* < 0.001), DLP (316.1 ± 101.3 vs 42.6 ± 8.9 mGy·cm, *p* < 0.001), and ED (4.43 ± 1.42 vs 0.60 ± 0.12 mSv, *p* < 0.001).

### Coronary stenosis result

Table 1 lists the overall coronary stenosis assessment of LD-HIR and LD-DLR groups at different levels, including assessments of ≥ 50% coronary stenosis and CAD-RADS scoring. Tables 2 and 3 summarize the coronary stenosis assessments of LD-HIR and LD-DLR groups in different vessels and segments, respectively.

**Table 1:** The overall coronary stenosis assessment of two low-dose CCTA groups at different levels

Type	Level	LD-HIR	LD-DLR	*p* value
≥ 50% stenosis	Per-segment (*n* = 712)			
	Sensitivity	0.91 [81/89] (0.76–0.96)	0.90 [80/89] (0.74–0.95)	1.00
	Specificity	0.94 [585/623] (0.91–0.96)	0.99 [615/623] (0.97–0.99)	< 0.001
	PPV	0.68 [81/119] (0.46–0.72)	0.91 [80/88] (0.76–0.95)	< 0.001
	NPV	0.99 [585/593] (0.97–0.99)	0.99 [615/624] (0.97–0.99)	1.00
	Accuracy	0.94 [666/712] (0.91–0.96)	0.98 [695/712] (0.96–0.99)	< 0.001
	AUC	0.92 (0.89–0.96)	0.94 (0.91–0.97)	< 0.01
	Per-vessel (*n* = 267)			
	Sensitivity	0.89 [50/56] (0.76–0.96)	0.88 [49/56] (0.74–0.94)	1.00
	Specificity	0.88 [186/211] (0.82–0.92)	0.97 [205/211] (0.93–0.99)	< 0.001
	PPV	0.67 [50/75] (0.48–0.75)	0.89 [49/55] (0.73–0.95)	< 0.01
	NPV	0.97 [186/192] (0.92–0.99)	0.97 [205/212] (0.92–0.98)	1.00
	Accuracy	0.88 [236/267] (0.83–0.92)	0.95 [254/267] (0.91–0.97)	< 0.001
	AUC	0.89 (0.84–0.93)	0.92 (0.88–0.97)	< 0.01
	Per-patient (*n* = 89)			
	Sensitivity	0.93 [28/30] (0.77–0.98)	0.90 [27/30] (0.73–0.97)	1.00
	Specificity	0.76 [45/59] (0.64–0.85)	0.95 [56/59] (0.85–0.98)	< 0.01
	PPV	0.67 [28/42] (0.51–0.79)	0.90 [27/30] (0.73–0.97)	< 0.05
	NPV	0.96 [45/47] (0.85–0.99)	0.95 [56/59] (0.85–0.98)	1.00
	Accuracy	0.82 [73/89] (0.73–0.89)	0.93 [83/89] (0.86–0.97)	< 0.01
	AUC	0.85 (0.78–0.92)	0.92 (0.86–0.99)	< 0.05
CAD-RADS scores	Per-segment (*n* = 712)			
	Accuracy	0.87 [617/712] (0.83–0.90)	0.94 [667/712] (0.91–0.96)	< 0.001
	Kappa	0.80 (0.72–0.85)	0.91 (0.86–0.94)	< 0.001
	Per-vessel (*n* = 267)			
	Accuracy	0.77 [205/267] (0.71–0.82)	0.88 [235/267] (0.83–0.92)	< 0.01
	Kappa	0.76 (0.68–0.82)	0.88 (0.82–0.92)	< 0.001
	Per-patient (*n* = 89)			
	Accuracy	0.61 [54/89] (0.50–0.70)	0.81 [72/89] (0.71–0.88)	< 0.01
	Kappa	0.67 (0.56–0.76)	0.85 (0.78–0.92)	< 0.001

Data is value [*n*/*N*] (adjusted 95% CI) or value (adjusted 95% CI)

*PPV* positive predictive value, *NPV* negative predictive value, *AUC* area under the curve, *CI* confidence interval, *LD-HIR* low-dose CCTA data reconstructed by the hybrid iterative reconstruction algorithm, *LD-DLR* low-dose CCTA data reconstructed by the deep learning reconstruction algorithm, *CAD-RADS* coronary artery disease-reporting and data system

**Table 2:** The coronary stenosis assessment of two low-dose CCTA groups in different vessels

Type	Level	LD-HIR	LD-DLR	*p* value
≥ 50% stenosis	RCA (*n* = 89)			
	Sensitivity	0.88 [14/16] (0.61–0.97)	0.88 [14/16] (0.61–0.97)	1.00
	Specificity	0.93 [68/73] (0.85–0.97)	0.96 [70/73] (0.88–0.99)	0.50
	PPV	0.74 [14/19] (0.50–0.89)	0.82 [14/17] (0.57–0.94)	0.70
	NPV	0.97 [68/70] (0.89–0.99)	0.97 [70/72] (0.90–0.99)	1.00
	Accuracy	0.92 [82/89] (0.84–0.96)	0.94 [84/89] (0.87–0.98)	0.50
	AUC	0.90 (0.81–0.99)	0.92 (0.83–1.00)	0.15
	LAD (*n* = 89)			
	Sensitivity	0.96 [25/26] (0.77–0.99)	0.92 [24/26] (0.74–0.98)	1.00
	Specificity	0.76 [48/63] (0.64–0.85)	0.95 [60/63] (0.86–0.98)	< 0.001
	PPV	0.62 [25/40] (0.47–0.76)	0.89 [24/27] (0.71–0.96)	< 0.05
	NPV	0.98 [48/49] (0.87–1.00)	0.97 [60/62] (0.88–0.99)	1.00
	Accuracy	0.82 [73/89] (0.73–0.89)	0.94 [84/89] (0.87–0.98)	< 0.01
	AUC	0.86 (0.80–0.93)	0.94 (0.88–1.00)	< 0.05
	LCX (*n* = 89)			
	Sensitivity	0.79 [11/14] (0.51–0.93)	0.79 [11/14] (0.51–0.93)	1.00
	Specificity	0.93 [70/75] (0.85–0.97)	1.00 [75/75] (1.00–1.00)	0.06
	PPV	0.69 [11/16] (0.43–0.86)	1.00 [11/11] (1.00–1.00)	0.06
	NPV	0.96 [70/73] (0.88–0.99)	0.96 [75/78] (0.89–0.99)	1.00
	Accuracy	0.91 [81/89] (0.83–0.95)	0.97 [86/89] (0.90–0.99)	0.06
	AUC	0.86 (0.74–0.97)	0.89 (0.78–1.00)	< 0.05
CAD-RADS scores	RCA (*n* = 89)			
	Accuracy	0.82 [73/89] (0.73–0.89)	0.90 [80/89] (0.82–0.95)	0.20
	Kappa	0.80 (0.71–0.89)	0.89 (0.81–0.96)	< 0.001
	LAD (*n* = 89)			
	Accuracy	0.66 [59/89] (0.56–0.75)	0.85 [76/89] (0.76–0.91)	< 0.01
	Kappa	0.72 (0.63–0.80)	0.89 (0.83–0.94)	< 0.001
	LCX (*n* = 89)			
	Accuracy	0.82 [73/89] (0.73–0.89)	0.89 [79/89] (0.80–0.94)	0.29
	Kappa	0.71 (0.59–0.84)	0.81 (0.70–0.92)	< 0.05

Data is value [*n*/*N*] (adjusted 95% CI) or value (adjusted 95% CI)

*RCA* right coronary artery, *LAD* left anterior descending, *LCX* left circumflex

**Table 3:** The coronary stenosis assessment of two low-dose CCTA groups in different segments.

Type	Level	LD-HIR	LD-DLR	*p* value
≥ 50% stenosis	Proximal (*n* = 267)			
	Sensitivity	0.92 [36/39] (0.78–0.97)	0.90 [35/39] (0.74–0.96)	1.00
	Specificity	0.94 [214/228] (0.90–0.96)	0.99 [226/228] (0.97–1.00)	< 0.001
	PPV	0.72 [36/50] (0.52–0.80)	0.95 [35/37] (0.81–0.99)	< 0.05
	NPV	0.99 [214/217] (0.96–1.00)	0.98 [226/230] (0.96–0.99)	1.00
	Accuracy	0.94 [250/267] (0.90–0.96)	0.98 [261/267] (0.95–0.99)	< 0.01
	AUC	0.93 (0.89–0.98)	0.94 (0.90–0.99)	0.36
	Mid (*n* = 178)			
	Sensitivity	0.93 [26/28] (0.76–0.98)	0.93 [26/28] (0.76–0.98)	1.00
	Specificity	0.91 [137/150] (0.85–0.95)	0.99 [148/150] (0.95–1.00)	< 0.01
	PPV	0.67 [26/39] (0.48–0.79)	0.93 [26/28] (0.75–0.98)	< 0.05
	NPV	0.99 [137/139] (0.94–1.00)	0.99 [148/150] (0.95–1.00)	1.00
	Accuracy	0.92 [163/178] (0.86–0.95)	0.98 [174/178] (0.94–0.99)	< 0.01
	AUC	0.92 (0.87–0.97)	0.96 (0.91–1.00)	< 0.001
	Distal (*n* = 267)			
	Sensitivity	0.86 [19/22] (0.61–0.95)	0.86 [19/22] (0.61–0.95)	1.00
	Specificity	0.96 [234/245] (0.91–0.98)	0.98 [241/245] (0.95–0.99)	< 0.05
	PPV	0.63 [19/30] (0.40–0.78)	0.83 [19/23] (0.58–0.94)	0.22
	NPV	0.99 [234/237] (0.96–1.00)	0.99 [241/244] (0.96–1.00)	1.00
	Accuracy	0.95 [253/267] (0.91–0.97)	0.97 [260/267] (0.94–0.99)	< 0.05
	AUC	0.91 (0.83–0.98)	0.92 (0.85–1.00)	< 0.01
CAD-RADS scores	Proximal (*n* = 267)			
	Accuracy	0.85 [227/267] (0.79–0.89)	0.93 [248/267] (0.88–0.96)	< 0.01
	Kappa	0.83 (0.76–0.88)	0.92 (0.87–0.96)	< 0.001
	Mid (*n* = 178)			
	Accuracy	0.84 [149/178] (0.78–0.88)	0.94 [167/178] (0.89–0.96)	< 0.01
	Kappa	0.81 (0.74–0.87)	0.95 (0.92–0.97)	< 0.001
	Distal (*n* = 267)			
	Accuracy	0.90 [241/267] (0.86–0.93)	0.94 [252/267] (0.91–0.97)	0.10
	Kappa	0.71 (0.55–0.82)	0.82 (0.70–0.91)	< 0.001

Data is value [*n*/*N*] (adjusted 95% CI) or value (adjusted 95% CI)

The primary findings were that LD-DLR showed significantly higher specificity, PPV, accuracy, and AUC for ≥ 50% stenosis at per-segment, per-vessel, and per-patient levels. Specifically, LD-DLR showed significantly higher specificity (0.99 vs 0.94), PPV (0.91 vs 0.68), accuracy (0.98 vs 0.94) and AUC (0.94 vs 0.92) at per-segment level (all *p* < 0.01); higher specificity (0.97 vs 0.88), PPV (0.89 vs 0.67), accuracy (0.95 vs 0.88) and AUC (0.92 vs 0.89) at per-vessel level (all *p* < 0.01); and higher specificity (0.95 vs 0.76), PPV (0.90 vs 0.67), accuracy (0.93 vs 0.82) and AUC (0.92 vs 0.85) at per-patient level (all *p* < 0.05), compared to LD-HIR. No significant differences were found in sensitivity and NPV between the two groups at three levels (all *p* > 0.05).

For ≥ 50% stenosis at different vessels and segments, LD-DLR achieved significantly higher specificity (0.95 vs 0.76), PPV (0.89 vs 0.62), accuracy (0.94 vs 0.82) and AUC (0.94 vs 0.86) at LAD-vessel level; higher AUC (0.89 vs 0.86) at LCX-vessel level; higher specificity (0.99 vs 0.94), PPV (0.95 vs 0.72) and accuracy (0.98 vs 0.94) at proximal-segment level; higher specificity (0.99 vs 0.91), PPV (0.93 vs 0.67), accuracy (0.98 vs 0.92) and AUC (0.96 vs 0.92) at mid-segment level; and higher specificity (0.98 vs 0.96), accuracy (0.97 vs 0.95) and AUC (0.92 vs 0.91) at distal-segment level than LD-HIR (all *p* < 0.05). No significant differences were observed in all six metrics at the RCA-vessel level; sensitivity and NPV at LAD-vessel level; sensitivity, specificity, PPV, NPV, and accuracy at LCX-vessel level; sensitivity, NPV, and AUC at proximal-segment level; sensitivity and NPV at mid-segment level; and sensitivity, PPV, and NPV at distal-segment level between the two groups (all *p* > 0.05).

**Fig. 1:**
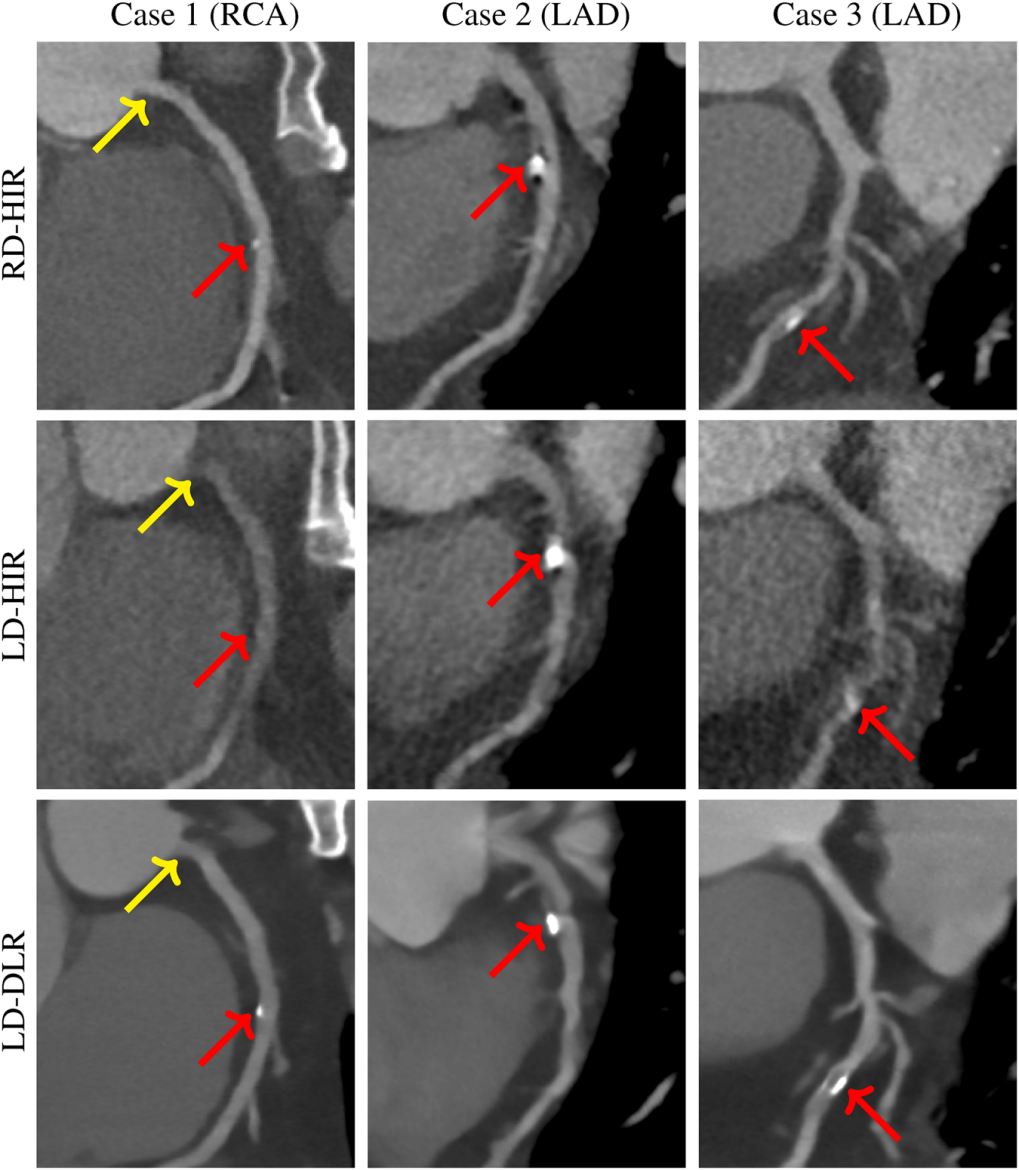
The CPR images of three patients in three groups. Case 1 shows RCA vessels from one 59-year-old male in RD-HIR, LD-HIR, and LD-DLR groups, and the CAD-RADS scores are 0 (negative), 1 (negative), and 0 (negative) in the proximal RCA marked by yellow arrows and 1 (negative), 0 (negative), and 1 (negative) in the mid RCA marked by red arrows. Case 2 shows the LAD vessel from one 62-year-old male in three groups, and the CAD-RADS scores are 2 (negative), 4 (positive), and 2 (negative) at proximal LAD marked by red arrows. Case 3 shows LAD vessels from another 62-year-old male in three groups, and the CAD-RADS scores are 2 (negative), 3 (positive), and 2 (negative) in the mid LAD marked by red arrows

For CAD-RADS scoring, LD-DLR obtained significantly higher accuracy (0.94 vs 0.87) and kappa (0.91 vs 0.80) at per-segment level, higher accuracy (0.88 vs 0.77) and kappa (0.88 vs 0.76) at per-vessel level, higher accuracy (0.81 vs 0.61) and kappa (0.85 vs 0.67) at per-patient level, higher kappa (0.89 vs 0.80) at RCA-vessel level, higher accuracy (0.85 vs 0.66) and kappa (0.89 vs 0.72) at LAD-vessel level, higher kappa (0.81 vs 0.71) at LCX-vessel level, higher accuracy (0.93 vs 0.85) and kappa (0.92 vs 0.83) at proximal-segment level, higher accuracy (0.94 vs 0.84) and kappa (0.95 vs 0.81) at mid-segment level, and higher kappa (0.82 vs 0.71) at distal-segment level than LD-HIR (all *p* < 0.05). There were no significant differences in accuracy at RCA-vessel, LCX-vessel, and distal-segment levels between the two groups (all *p* > 0.05). Figure 1 shows the curved planar reformation (CPR) images of RD-HIR, LD-HIR, and LD-DLR groups.

Additionally, in the exploratory subgroup analysis of nine patients with high CACS, LD-DLR and LD-HIR groups yielded comparable results for ≥ 50% stenosis (sensitivity, PPV, and accuracy: all 1.00 vs 1.00) and CAD-RADS scoring (accuracy: 0.78 vs 0.78; kappa: 0.36 vs 0.27).

### CT-FFR result

**Fig. 2:**
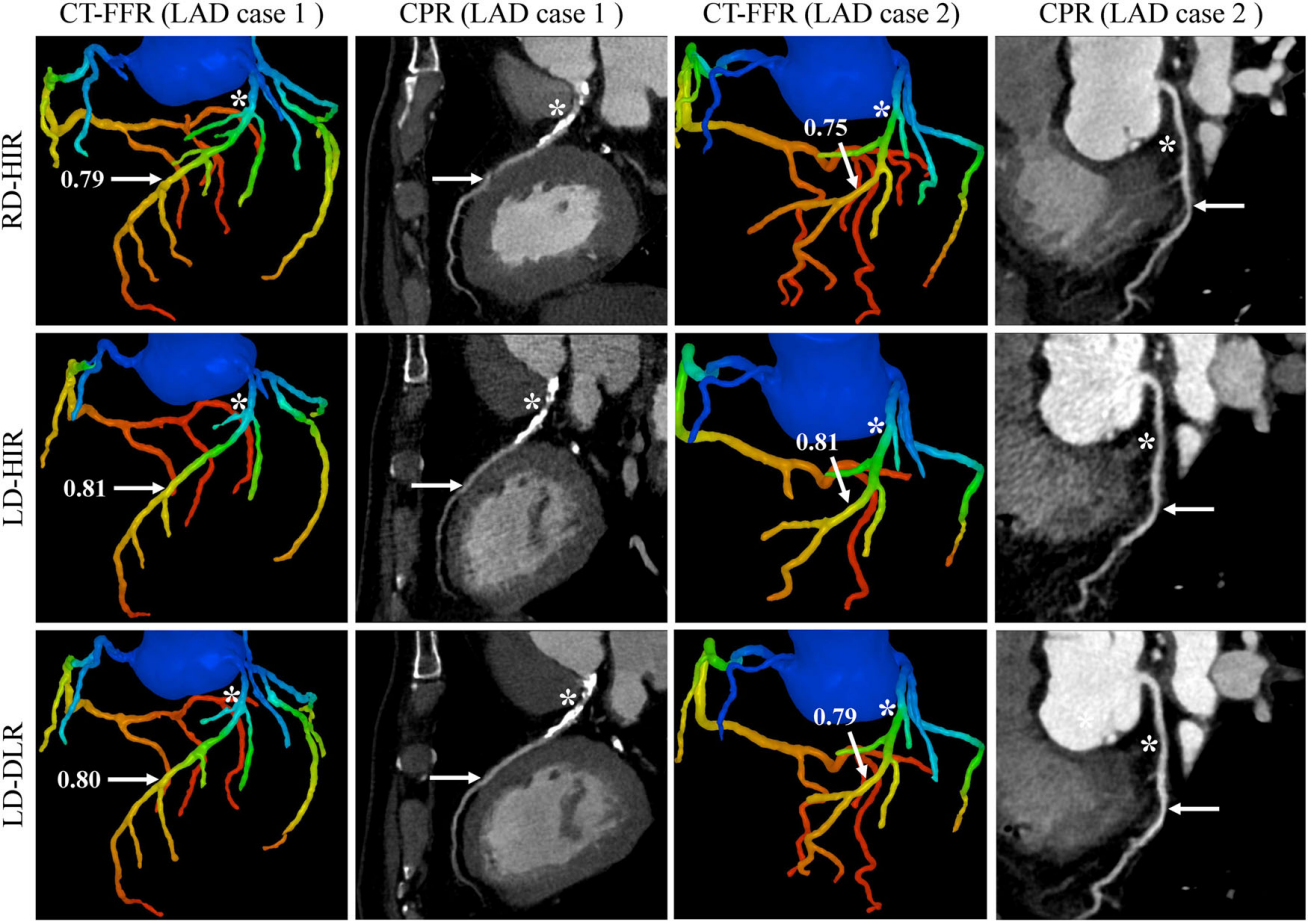
The CT-FFR and CPR images of two patients in three groups. Case 1 shows LAD vessels from one 65-year-old male in the RD-HIR, LD-HIR, and LD-DLR groups, and the CT-FFR values are 0.79 (positive), 0.81 (negative), and 0.80 (positive) in the LAD vessel marked by white arrows. Case 2 shows LAD vessels from one 62-year-old male in three groups, and the CT-FFR values are 0.75 (positive), 0.81 (negative), and 0.79 (positive) in the LAD vessel marked by white arrows. The white asterisk (*) indicates the location of stenosis, and the white arrow represents the CT-FFR value measured 20 mm distal to the stenosis

Table 4 exhibits the CT-FFR results of the LD-HIR and LD-DLR groups at various levels. LD-DLR showed significantly higher sensitivity (0.90 vs 0.50), NPV (0.99 vs 0.96), accuracy (0.97 vs 0.94), and AUC (0.94 vs 0.74) at the per-vessel level, and higher AUC at the LAD-vessel level (0.94 vs 0.71) and the LCX-vessel level (0.99 vs 0.67) compared with LD-HIR (all *p* < 0.05). No significant differences were observed in specificity and PPV at per-vessel, all six metrics at per-patient and RCA-vessel levels, and sensitivity, specificity, PPV, NPV, and accuracy at LAD-vessel and LCX-vessel levels (all *p* > 0.05). Figure 2 shows the CT-FFR and CPR images of RD-HIR, LD-HIR, and LD-DLR.

**Table 4:** The CT-FFR results of two low-dose CCTA groups at various levels

Level	LD-HIR	LD-DLR	*p* value
Per-vessel (*n* = 267)			
Sensitivity	0.50 [10/20] (0.32–0.70)	0.90 [18/20] (0.67–0.99)	< 0.05
Specificity	0.98 [241/247] (0.95–0.99)	0.98 [242/247] (0.95–0.99)	1.00
PPV	0.62 [10/16] (0.30–0.78)	0.78 [18/23] (0.60–0.90)	0.31
NPV	0.96 [241/251] (0.92–0.98)	0.99 [242/244] (0.95–1.00)	< 0.05
Accuracy	0.94 [251/267] (0.90–0.97)	0.97 [260/267] (0.94–0.99)	< 0.05
AUC	0.74 (0.63–0.85)	0.94 (0.87–1.00)	< 0.01
Per-patient (*n* = 89)			
Sensitivity	0.71 [10/14] (0.44–0.89)	0.93 [13/14] (0.63–0.99)	0.38
Specificity	0.95 [71/75] (0.87–0.98)	0.96 [72/75] (0.88–0.99)	1.00
PPV	0.71 [10/14] (0.44–0.89)	0.81 [13/16] (0.55–0.94)	0.67
NPV	0.95 [71/75] (0.87–0.98)	0.99 [72/73] (0.91–1.00)	0.37
Accuracy	0.91 [81/89] (0.83–0.95)	0.96 [85/89] (0.89–0.98)	0.29
AUC	0.83 (0.71–0.96)	0.94 (0.87–1.00)	0.15
RCA (*n* = 89)			
Sensitivity	0.67 [4/6] (0.27–0.92)	0.83 [5/6] (0.37–0.98)	1.00
Specificity	0.96 [80/83] (0.89–0.99)	0.98 [81/83] (0.91–0.99)	1.00
PPV	0.57 [4/7] (0.23–0.86)	0.71 [5/7] (0.33–0.93)	1.00
NPV	0.98 [80/82] (0.91–0.99)	0.99 [81/82] (0.92–1.00)	1.00
Accuracy	0.94 [84/89] (0.87–0.98)	0.97 [86/89] (0.90–0.99)	0.50
AUC	0.82 (0.61–1.00)	0.90 (0.74–1.00)	0.28
LAD (*n* = 89)			
Sensitivity	0.45 [5/11] (0.20–0.73)	0.91 [10/11] (0.56–0.99)	0.13
Specificity	0.96 [75/78] (0.89–0.99)	0.97 [76/78] (0.90–0.99)	1.00
PPV	0.62 [5/8] (0.28–0.87)	0.83 [10/12] (0.52–0.96)	0.35
NPV	0.93 [75/81] (0.84–0.97)	0.99 [76/77] (0.91–1.00)	0.12
Accuracy	0.90 [80/89] (0.82–0.95)	0.97 [86/89] (0.90–0.99)	0.11
AUC	0.71 (0.55–0.86)	0.94 (0.85–1.00)	< 0.05
LCX (*n* = 89)			
Sensitivity	0.33 [1/3] (0.04–0.85)	1.00 [3/3] (1.00–1.00)	0.50
Specificity	1.00 [86/86] (1.00–1.00)	0.99 [85/86] (0.92–1.00)	1.00
PPV	1.00 [1/1] (1.00–1.00)	0.75 [3/4] (0.24–0.97)	1.00
NPV	0.98 [86/88] (0.91–0.99)	1.00 [85/85] (1.00–1.00)	0.50
Accuracy	0.98 [87/89] (0.91–0.99)	0.99 [88/89] (0.92–1.00)	1.00
AUC	0.67 (0.34–0.99)	0.99 (0.98–1.00)	< 0.05

Data is value [*n*/*N*] (adjusted 95% CI) or value (adjusted 95% CI)

### Image quality result

**Fig. 3:**
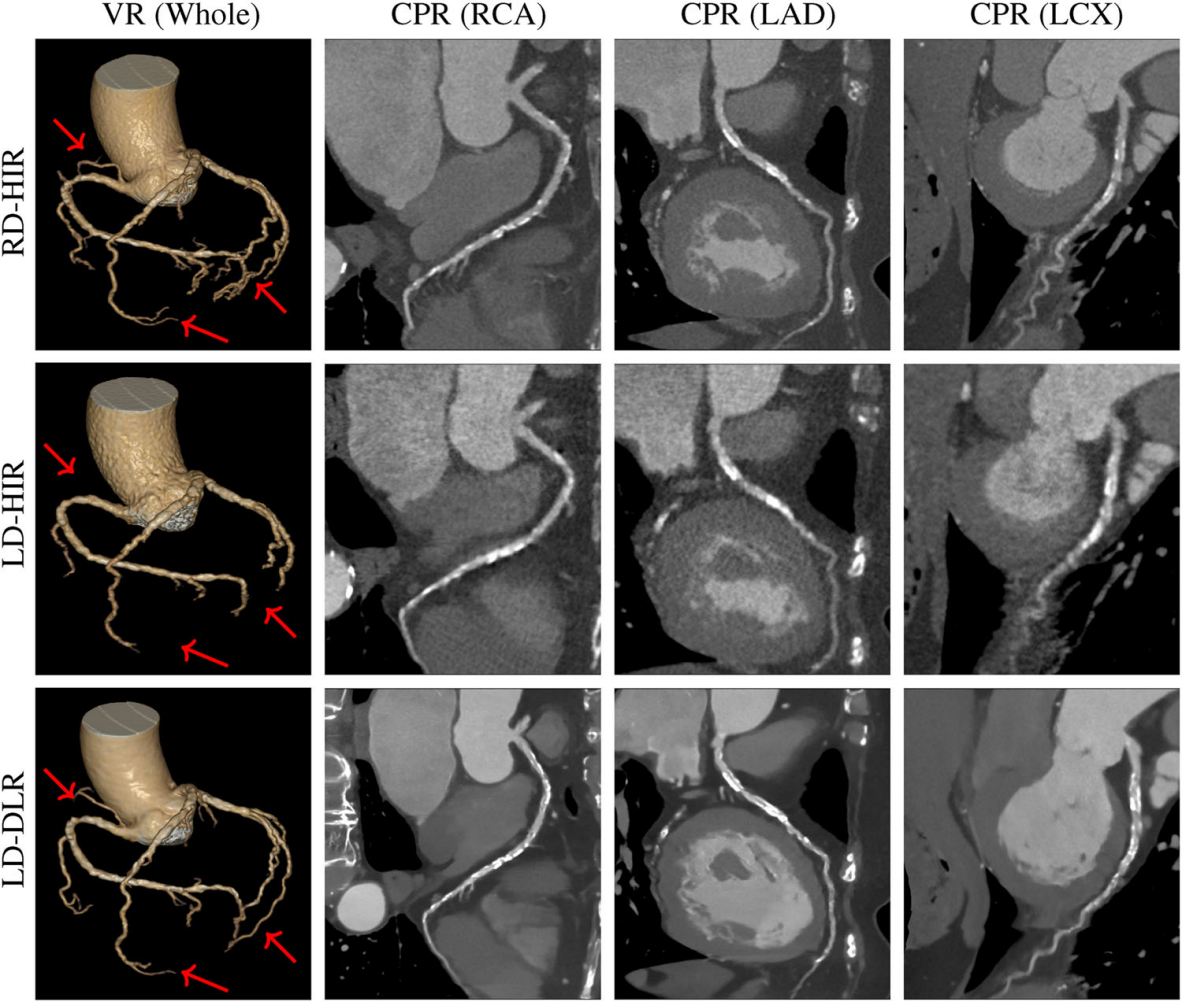
The VR and the CPR images of a 69-year-old female with diffuse calcification stenosis (all segments ≥ 50% stenosis) in three groups. The LD-DLR images demonstrate superior visualization compared to LD-HIR. Specifically, in the VR images, LD-DLR recovers many subtle vascular branches (red arrows) that are lost in LD-HIR. In the CPR images, LD-DLR provides sharper vessel edges and clearer definition of calcified plaques across all segments. The visual quality of LD-DLR is comparable to RD-HIR in the RCA, LCX, and the proximal/mid LAD, and is superior to RD-HIR in the distal LAD segment

Table 5 summarizes the subjective IQS of RD-HIR, LD-HIR, and LD-DLR groups. The IQS of LD-HIR is significantly lower than both RD-HIR and LD-DLR in all segments (all adjusted *p* < 0.001). No significant difference was found in IQS between LD-DLR and RD-HIR in all segments (all adjusted *p* > 0.05). Inter-rater agreement between the two radiologists for IQS assessment was excellent across all segments, with kappa values all exceeding 0.82. Figure 3 exhibits the whole coronary volume rendering (VR) and CPR images of three groups.

**Table 5:** The IQS of three groups in various segments

Segments	RD-HIR	LD-HIR	LD-DLR	*p* value
RCA (*n* = 89)				
Proximal	4.6 ± 0.5	3.6 ± 0.6	4.7 ± 0.5	< 0.001
Mid	4.6 ± 0.5	3.6 ± 0.6	4.7 ± 0.5	< 0.001
Distal	4.5 ± 0.5	3.5 ± 0.6	4.6 ± 0.5	< 0.001
LAD (*n* = 89)				
Proximal	4.5 ± 0.5	3.6 ± 0.6	4.6 ± 0.5	< 0.001
Mid	4.3 ± 0.5	3.3 ± 0.6	4.4 ± 0.6	< 0.001
Distal	4.2 ± 0.5	3.2 ± 0.6	4.4 ± 0.6	< 0.001
LCX (*n* = 89)				
Proximal	4.3 ± 0.5	3.4 ± 0.6	4.5 ± 0.5	< 0.001
Distal	4.1 ± 0.6	3.2 ± 0.5	4.3 ± 0.6	< 0.001

Data is mean ± SD

*RD-HIR* routine-dose CCTA data reconstructed by the hybrid iterative reconstruction algorithm

**Fig. 4:**
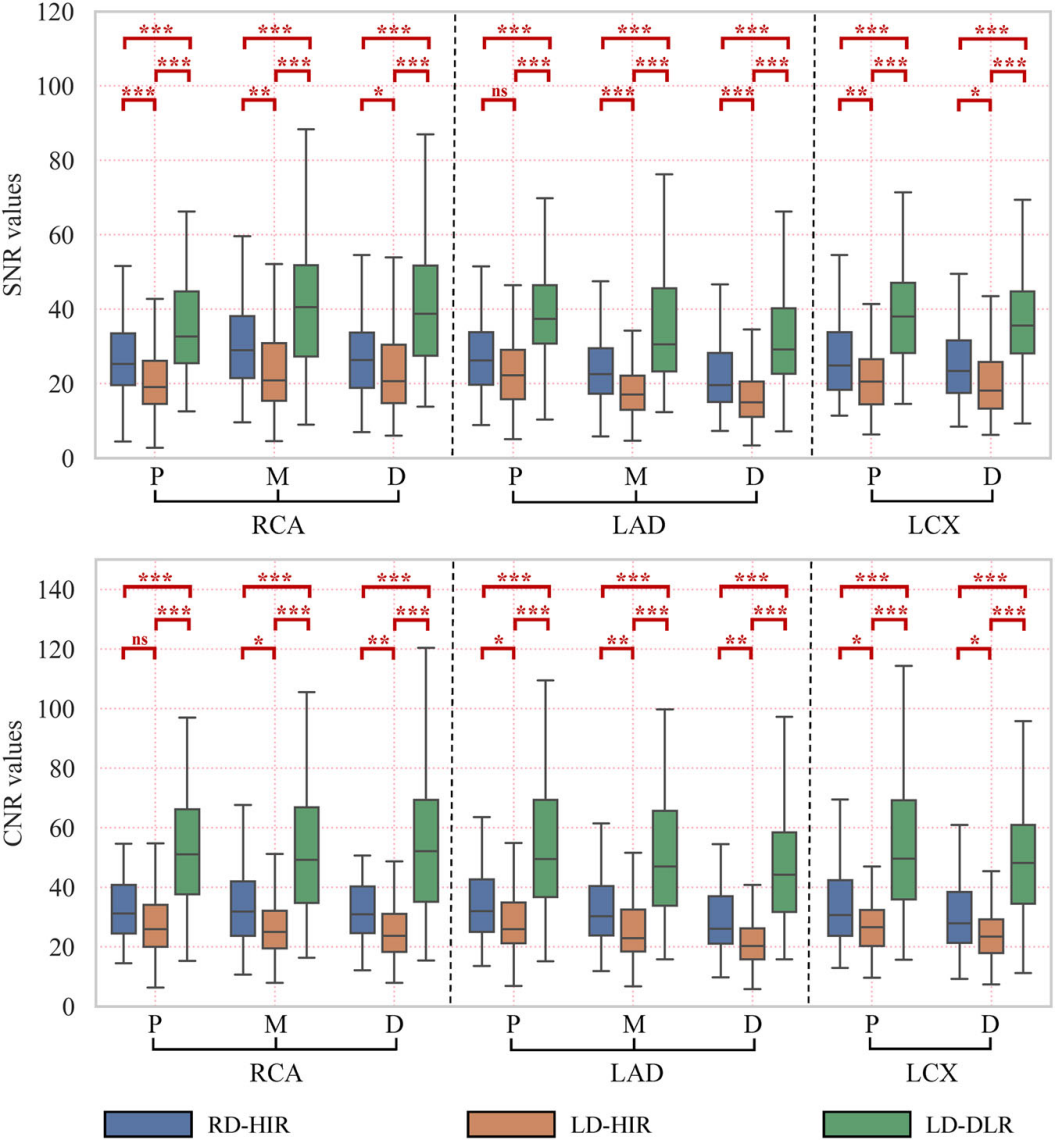
The boxplot diagrams of segment-level SNR and CNR in RD-HIR, LD-HIR, and LD-DLR groups. The symbols “P”, “M”, and “D” mean proximal, mid, and distal segments. The “*”, “**” and “***” represent 0.01 ≤ adjusted *p* < 0.05, 0.001 ≤ adjusted *p* < 0.01 and adjusted *p* < 0.001, and “ns” means no significance

Figure 4 shows boxplot diagrams of SNR and CNR in RD-HIR, LD-HIR, and LD-DLR groups. LD-DLR demonstrated significantly higher SNR and CNR than both RD-HIR and LD-HIR in all segments (all adjusted *p* < 0.001). No significant difference was observed between RD-HIR and LD-HIR in SNR of the proximal LAD and in CNR of the proximal RCA (both adjusted *p* = 0.06). However, LD-HIR showed significantly lower SNR and CNR than RD-HIR in other segments (all adjusted *p* < 0.05). At segment-level (*n* = 712), SNR was 27.8 ± 14.9 vs 21.6 ± 11.2 vs 39.8 ± 20.7, and CNR was 32.9 ± 14.4 vs 26.6 ± 12.0 vs 54.7 ± 29.6 for RD-HIR, LD-HIR, and LD-DLR groups (all adjusted *p* < 0.001).

## Discussion

This study tested the feasibility of a 60 kVp CCTA protocol integrated with a vendor-specific DLR algorithm, aiming to reduce radiation and contrast agent doses and maintain the consistent performance of coronary stenosis assessments and CT-FFR values with the conventional 100 kVp CCTA protocol.

Compared to previous low tube-voltage CCTA protocols, the 60 kVp CCTA protocol combined with DLR in this study achieved a higher mean segment-level SNR (39.8) and CNR (54.7), alongside a lower mean ED (0.60 mSv). For instance, the mean vessel-level SNR and CNR were 30.5 and 27.8 in the 80 kVp CCTA protocol for patients with BMI < 30 kg/m^2^ [9], which were 35.3 and 47.6 in the 70 kVp CCTA protocol for patients with BMI < 26 kg/m^2^ [11]. Furthermore, our study provided a more detailed and comprehensive evaluation by conducting assessments at the segment-, vessel-, and patient-levels, whereas the previous 80 kVp and 70 kVp protocols primarily performed qualitative and quantitative analyses at the vessel-level only. Additionally, the observed radiation dose reduction of 86.5% exceeds theoretical predictions based solely on tube voltage change alone, as these theoretical models often overlook key system hardware factors. As Greffier et al noted, the extent of dose reduction from kV changes can be influenced by the specific filtration design of the CT scanner [18]. Consequently, the lower-energy 60-kVp beam is disproportionately attenuated by the scanner’s filter due to the photoelectric effect, which drastically reduces the effective photon flux reaching the patient.

Previous studies have employed various low-dose contrast agent protocols. For instance, Caruso et al reported using a mean contrast dose of 44.8 mL in a 70 kVp protocol for patients with BMI ≤ 30 kg/m² [9], while Ren et al and Feng et al utilized even lower doses of 32.1 mL and 28 mL in non-obese populations, respectively [19, 20]. In the present study, to ensure a high examination success rate, we opted for a conservative approach and avoided overly aggressive contrast dose reduction. Future investigations may explore the use of lower-concentration contrast agents or diluted contrast-saline mixtures as a promising avenue for further dose minimization. Moreover, the double-low-dose protocol in this study required modifications only to the tube voltage and the contrast agent’s total volume and flow rate, with all other scanning parameters remaining unchanged. This simplicity facilitates seamless integration into established clinical workflows without necessitating additional training.

This study has several limitations. First, it focused exclusively on the consistency analysis of coronary stenosis and CT-FFR between 60 kVp and 100 kVp CCTA protocols, and the diagnostic performance of the 60 kVp protocol for other coronary pathologies requires further evaluation. Second, the analysis was limited to differentiating mild from moderate-to-severe stenosis. The accuracy of luminal assessment under this protocol may be compromised in patients with severe calcification, warranting additional investigation. Third, the absence of an invasive reference standard such as digital subtraction angiography (DSA) must be noted. However, as the diagnostic accuracy of routine-dose CCTA for coronary stenosis is well-established [21–23], RD-HIR was considered a reliable reference for comparing LD-HIR and LD-DLR in this study. Similarly, since invasive FFR was not available, this study could only demonstrate that the 60 kVp protocol produced CT-FFR values consistent with the 100 kVp protocol. However, CT-FFR derived from routine-dose scans has been validated against invasive FFR with a mean accuracy of 0.84 [24] and used in long-term risk assessment for adverse cardiovascular events [25]. CT-FFR values from the 60 kVp protocol still require direct validation against invasive FFR. Fourth, residual contrast from the first scan might affect the second scan. However, as the second scan served as a reference group, any potential overestimation of CT values, SNR, and CNR in this group would not fundamentally affect the evaluation of image quality improvement achieved by the DLR algorithm. Fifth, the subgroup with high CACS scores comprised only 9 patients, lacking the statistical power to conduct meaningful tests. The diagnostic validity requires further validation in larger patient cohorts in the future. Sixth, the DLR algorithm and platform used are vendor-specific and proprietary. However, given the similarity in the underlying principles of deep learning-based reconstruction algorithms, the findings of this study are likely generalizable to a wider range of CT systems. Seventh, the reference standard in this study was the routine-dose protocol with HIR, and the routine-dose protocol with DLR was not included for comparison. Finally, the study population was recruited from a single country, which may limit the generalizability of the findings due to potential demographic and racial biases.

In conclusion, this study demonstrated that a 60 kVp double-low-dose CCTA protocol combined with the DLR algorithm markedly reduced radiation dose by 86.5% compared to the conventional 100 kVp CCTA protocol. The proposed 60 kVp protocol maintained or even improved image quality while preserving the highly consistent performance of coronary stenosis and CT-FFR assessments with the 100 kVp protocol (all AUC ≥ 0.89).

## Supplementary information

Supplementary information

## Data Availability

All patient data in this work is not publicly available. For any inquiries or additional information, the corresponding author can be contacted.

## Abbreviations

AUC: Area under the curve
BMI: Body mass index
CAD: Coronary artery disease
CAD-RADS: CAD-reporting and data system
CCTA: Coronary computed tomography angiography
CI: Confidence interval
CNR: Contrast-noise-ratio
CPR: Curved planar reformation
CTDIvol: Volume CT dose index
CT-FFR: Computed tomography-derived fractional flow reserve
DLP: Dose-length product
DLR: Deep learning reconstruction
ED: Effective dose
GEE: Generalized estimating equations
HIR: Hybrid iterative reconstruction
HR: Heart rate
IQS: Image quality score
LAD: Left anterior descending
LCX: Left circumflex
LD-DLR: Low-dose CCTA data reconstructed with the DLR algorithm
LD-HIR: Low-dose CCTA data reconstructed with the HIR algorithm
NPV: Negative predictive value
PPV: Positive predictive value
RCA: Right coronary artery
RD-HIR: Routine-dose CCTA data reconstructed by the HIR algorithm
ROC: Receiver operating characteristic
ROI: Region of interest
SD: Standard deviation
SNR: Signal-noise-ratio
VR: Volume rendering
