# Supplementary material for: Clinical feasibility test of 60 kVp double-low-dose coronary CT angiography with a deep learning reconstruction algorithm
Source: Insights Imaging. 2026 Feb 10;17:41. doi: 10.1186/s13244-026-02223-6 (PMC12891270; doi:10.1186/s13244-026-02223-6)
Supplement: Supplementary file 1 — Supplementary information [file 13244_2026_2223_MOESM1_ESM.pdf]

Clinical feasibility test of 60 kVp double-low-dose coronary CT angiography with a deep learning reconstruction algorithm

ELECTRONIC SUPPLEMENTARY MATERIAL

Table S1. Demographic data and clinical information of all participants

Characteristic	Overall (n = 89)
Female	49.4% (44/89)
Age (years)	59.9 ± 13.2 (17-80)
BMI (kg/m^2)	23.1 ± 3.3 (14.6-31.2)
HR (bpm)	76.8 ± 12.2 (50-104)
HRv (bpm)	7.8 ± 1.8 (5-13)
Chest tightness and pain	34.8% (31/89)
Hypertension	32.6% (29/89)
Diabetes	5.6% (5/89)
Hyperlipidemia	18.0% (16/89)
Smoking	30.3% (27/89)
Drinking	25.8% (23/89)
Family history of CAD	7.9% (7/89)
Arrhythmia	13.5% (12/89)
Atrial fibrillation	4.5% (4/89)
History of myocardial infarction	4.5% (4/89)

Data are mean ± standard deviation (range) or % (n/N)

BMI body mass index, *HR* heart rate, *HRv* heart rate variability, *bpm* beats per minute, *CAD* coronary artery disease
